# Advanced Science and Technology of Polymer Matrix Nanomaterials

**DOI:** 10.3390/ma17020461

**Published:** 2024-01-18

**Authors:** Peijiang Liu, Liguo Xu, Jinlei Li, Jianping Peng, Zibao Jiao

**Affiliations:** 1 Reliability Physics and Application Technology of Electronic Component Key Laboratory, The Fifth Electronics Research Institute of the Ministry of Industry and Information Technology, Guangzhou 510610, China; cz2343222@163.com; 2 College of Light Chemical Industry and Materials Engineering, Shunde Polytechnic, Foshan 528333, China; 3 Science and Technology on Space Physics Laboratory, Beijing 100076, China; 4 School of Materials Science and Hydrogen Energy, Foshan University, Foshan 528000, China; 5 Jiangsu Urban and Rural Construction Vocational College, Changzhou 213147, China

## 1. Introduction

The advanced science and technology of polymer matrix nanomaterials are rapidly developing fields that focus on the synthesis, characterization, and application of nanomaterials in polymer matrices [1,2,3,4,5]. Combined together as an interdisciplinary area, they integrate principles from materials science, chemistry, physics, and engineering to create novel materials with enhanced properties.

In recent years, researchers have achieved significant advancements in the design and fabrication of polymer matrix nanocomposites. These materials consist of polymer matrices that are reinforced or modified with nanoscale fillers such as nanoparticles, nanofibers, and nanotubes [6]. The incorporation of these nanofillers into the polymer matrix leads to improved mechanical, electrical, thermal, and optical properties.

A key challenge in this field is achieving uniform dispersion and strong interfacial interactions between the polymer matrix and the nanofillers. Various techniques, including melt mixing, solution blending, templated synthesis, and in situ polymerization, have been employed to overcome this challenge [7,8,9,10,11,12]. These techniques enable precise control over the distribution of nanofillers within the polymer matrix, resulting in materials with tailored properties.

Polymer matrix nanomaterials find applications in a wide range of industries, including aerospace, electronics, energy, automotive, and biomedical [13,14,15,16,17,18,19]. For example, in the biomedical field, polymer matrix nanomaterials can be used for drug delivery systems, tissue engineering scaffolds, and biosensors [20]. In aerospace applications, nanocomposites offer lightweight and high-strength alternatives to conventional materials, leading to improved fuel efficiency and reduced emissions [21].

Furthermore, advances in characterization techniques such as transmission electron microscopy (TEM), X-ray diffraction (XRD), atomic force microscopy (AFM), and thermal analysis methods have allowed researchers to study the structure–property relationships of polymer matrix nanomaterials on the nanoscale [22,23,24]. These techniques provide valuable insights into the orientation, dispersion, and interfacial interactions of nanofillers within the polymer matrix.

In summary, the advanced science and technology of polymer matrix nanomaterials are rapidly evolving fields that offer exciting prospects for the development of innovative materials with enhanced properties. The precise control of nanofiller dispersion within polymer matrices, along with the use of advanced characterization techniques, enables researchers to tailor the properties of these materials for various applications [25,26].

In dealing with these challenging aspects of polymer matrix nanomaterials, the goal of the present Special Issue is to introduce the current knowledge on the designs, synthesis processes, characterizations, properties, and applications of polymer matrix nanomaterials.

## 2. Contributions

Kim et al. [27] prepared polypropylene composites filled with randomly dispersed graphene nanoplatelets (GNPs) and a segregated GNP network. Theoretical and experimental investigations were conducted to explore the enhancements in the thermal and electrical conductivities of the composites achieved through the selective localization of GNP fillers using a segregated structure and the formation of a conductive network.

Zhu et al. [28] successfully synthesized a series of molecular wires based on [2.2]paracyclophane-1,9-dienes and then elucidated the influence of transannular π–π interaction on carrier transport in these wires using the STM break junction technique. Both the current–voltage characteristics and single-molecule conductance could be systematically adjusted through the transannular π–π interaction.

Most of the current research on agitator design primarily focuses on enhancing solid–liquid mixing efficiency and homogeneity, while neglecting the stability of the liquid level. He et al. [29] utilized computational fluid dynamics modeling to compare the performance of two types of rotor–stator agitators in solid–liquid mixing operations. The evaluation included aspects such as power consumption, homogeneity, and liquid-level stability. The results indicated that the cross structure rotor–stator agitator achieved a significantly lower standard deviation of particle concentration *σ* of 0.15 compared to the A200 agitators, with a 42% reduction.

Yeh et al. [30] studied the impact of hydrophilic and hydrophobic mesoporous silica particles (MSPs) on the dielectric properties of composite membranes derived from polyester imide (PEI). The study revealed a clear trend in the dielectric constant of the membranes: PEI containing hydrophilic MSPs > PEI > PEI containing hydrophobic MSPs.

Yin et al. [31] conducted a numerical investigation on the displacement of immiscible fluid in porous media using the lattice Boltzmann method. The results demonstrated that the wetting gradient can control the displacement pattern and efficiency. By introducing a wetting gradient in porous media, the stability of the flow front can be enhanced. This finding was confirmed across a wide range of parameters, including different wetting gradients, capillary numbers, viscosity ratios, and porosities.

Arputharaj et al. [32] provided a comprehensive review of biopolymeric nanoparticles developed for biomedical applications, such as drug delivery, imaging, and tissue engineering. The authors also discussed important fabrication techniques, along with the challenges and future perspectives in this field. It is crucial to address the interaction between nanoparticles and the immune system, as well as their elimination from the human body, in future studies.

Pozdnyakov et al. [33] conducted an analysis of the structural characteristics and direct current (DC) electrical conductivity of organic–inorganic nanocomposites composed of thermoelectric Te0 nanoparticles and poly(1-vinyl-1,2,4-triazole). The findings revealed that the DC electrical conductivity of nanocomposites containing 2.8 and 4.3 wt% Tellurium at 80 °C exceeded the conventional boundary of 10^−10^ S/cm, separating dielectrics and semiconductors.

Bekeschus et al. [34] generated unilamellar vesicles using 1-palmitoyl-2-oleoyl-glycero-3-phosphocholine (POPC) and 1-palmitoyl-2-oleoyl-sn-glycero-3-phospho-L-serine (POPS). These vesicles were then incubated with pristine, carboxylated, or aminated polystyrene spheres to form lipid coronas around the particles. This study, for the first time, demonstrated the influence of different lipid types on differently charged micro- and nanoplastic particles and the resulting biological implications.

Acierno et al. [35] conducted a study to examine the impact of different types of nanoparticles on the UV weathering resistance of polyurethane (PU) treatment in polyester-based fabrics. The findings revealed that incorporating nanoparticles into impregnated fabrics did not significantly hinder polymer degradation following UV exposure. However, the nanoparticles appeared to enhance the reinforcement of PU polymers within the textile structure, thereby improving the overall mechanical strength, particularly after UV exposure.

Xie et al. [36] employed a simple solvent-handling method to fabricate silylated GO/FeSiAl epoxy composites. They subsequently explored the microwave absorption properties and thermal conductivity. Remarkably, it was observed that these composites achieved a reflection loss of up to −48.28 dB and an effective range of 3.6 GHz when operating at frequencies between 2.575 and 2.645 GHz, with a modest thickness of just 2 mm. These results underscored the high absorption performance of the composites, making them suitable for packaging 5G base stations. 

The Guest Editors would like to extend their congratulations to all of the authors whose remarkable results have been published in this Special Issue. The papers presented here are expected to greatly contribute to the research community’s understanding of the current status and trends in the advanced science and technology of polymer matrix nanomaterials. Moreover, the Guest Editors cordially invite all scientists working in this field to submit innovative articles for consideration in the second edition of the Special Issue on “Advanced Science and Technology of Polymer Matrix Nanomaterials (2nd Edition)”.
